# GITAR: An Open Source Tool for Analysis and Visualization of Hi-C Data

**DOI:** 10.1016/j.gpb.2018.06.006

**Published:** 2018-12-13

**Authors:** Riccardo Calandrelli, Qiuyang Wu, Jihong Guan, Sheng Zhong

**Affiliations:** 1 Department of Bioengineering, University of California San Diego, La Jolla, CA 92093, USA; 2 Department of Computer Science and Technology, Tongji University, Shanghai 200092, China

**Keywords:** Chromatin interaction, Pipeline, Hi-C data normalization, Topologically-associated domain, Processed Hi-C data library

## Abstract

Interactions between chromatin segments play a large role in functional genomic assays and developments in genomic interaction detection methods have shown interacting topological domains within the genome. Among these methods, Hi-C plays a key role. Here, we present the Genome Interaction Tools and Resources (GITAR), a software to perform a comprehensive Hi-C data analysis, including data preprocessing, normalization, and visualization, as well as analysis of **topologically-associated domains** (TADs). GITAR is composed of two main modules: (1) HiCtool, a Python library to process and visualize Hi-C data, including TAD analysis; and (2) processed data library, a large collection of human and mouse datasets processed using HiCtool. HiCtool leads the user step-by-step through a **pipeline**, which goes from the raw Hi-C data to the computation, visualization, and optimized storage of intra-chromosomal contact matrices and TAD coordinates. A large collection of standardized processed data allows the users to compare different datasets in a consistent way, while saving time to obtain data for visualization or additional analyses. More importantly, GITAR enables users without any programming or bioinformatic expertise to work with Hi-C data. GITAR is publicly available at http://genomegitar.org as an open-source software.

## Introduction

Genomes are more than linear sequences, with DNA folded up into elaborate physical structures that allow extreme spatial compactness of the genetic material and play also an important role in epigenetic regulation [1], [2]. During the past fifteen years, several techniques have been developed to explore the genome architecture, such as Chromosome Conformation Capture (3C) [3], Circular Chromosome Conformation Capture (4C) [4], Chromosome Conformation Capture Carbon Copy (5C) [5], Hi-C [6], and chromatin interaction analysis by paired-end tag sequencing (ChIA-PET) [7]. Among them, Hi-C is one of the most important techniques, allowing for the first time a genome-wide mapping of chromatin interactions. In 2012, Dixon et al. [8] exploited Hi-C data to identify local chromosomal interacting domains that they named “topological domains”. These megabase-sized structures appear to be widespread along the genome, conserved between mice and humans, and stable across different cell types [8]. Later in 2014, Rao and colleagues generated higher resolution contact maps by performing Hi-C in intact nuclei (*in situ* Hi-C) [9].

Several steps are needed to process Hi-C data. First, raw data are subjected to preprocessing, including read alignment and filtering to remove low-quality mapped reads, PCR duplicates, as well as non-informative pairs. Some strategies could be implemented to improve the mapping outcome, such as pre-truncating reads, iterative mapping, allowing split alignments, and splitting if not mapped as mentioned by Ay and Noble [10]. Next, contact matrices are generated. To do so, the linear genome is divided into loci of a definite size, to create the matrix rows and columns. Given that, the entries of the matrix contain the number of contacts observed among each pair of loci. Contact matrices are then normalized to remove major systematic biases introduced during the experiment. These matrices are usually visualized using heatmaps, with pixel intensity proportional to the contact count within each entry.

Currently, there are several published tools to process and analyze Hi-C data (Table 1). While most of the software are focused just on specific parts of the analysis, only few of them present the entire Hi-C data processing pipeline mentioned above. The former usually contain additional features related to the specific parts of analysis, but it is often difficult for the users to provide the input data requested, especially for those who are not very familiar with Hi-C data analysis or programming in general. Instead, a single comprehensive tool would provide all the means needed to carry out the entire analysis, thus helpful in circumventing the difficulty related to data integration. This is particularly valuable, when different tasks are performed by several software and the user needs to figure out which tools to use and if the data are in the specific format required by each of them. A few software are comprehensive and allow for data preprocessing, contact matrix normalization, visualization, and topologically-associated domain (TAD) analysis. In addition, they may include other functions such as annotation of genomic features and chromatin loops over interactions, or interactive Hi-C map visualization. However, input file generation could be left to the user (for example, tracks such as GC content or mappability) and programming skills are often required to exploit the full capabilities of these tools.

**Table 1:** **Summary of Hi-C data analysis software and related features**

**Software**	**Data preprocessing**	**Data normalization**	**Data visualization**	**TAD analysis**	**Processed data library**	**Ref.**
chromoR		+				[11]
diffHiC	+	+	+	+		[12]
HiCapp	+	+				[13]
HiC-bench	+	+	+	+		[14]
HiCdat	+	+	+	+		[15]
HiC-inspector	+		+			
hiclib	+	+	+			[16]
HiCNorm		+				[17]
Hi-Corrector		+				[18]
Hicpipe		+				[19]
HiCPlotter			+			[20]
HiC-Pro	+	+				[21]
HiCseg				+		[22]
HiCUP	+					[23]
HiFive		+	+			[24]
HiGlass			+			[25]
HIPPIE	+					[26]
HiTC		+	+	+		[27]
HOMER		+	+	+		
HubPredictor				+		[28]
Juicer	+	+	+^*^	+	+	[29]
TADbit	+	+	+	+		[30]
TADtree				+		[31]
**GITAR**	**+**	**+**	**+**	**+**	**+**	**Current study**

*Note*: * indicates that the data visualization in Juicer is provided by the “associated” visualization software Juicebox [32], [33]. There are no publications associated with HiC-inspector and HOMER, which can be accessed at https://github.com/HiC-inspector and http://homer.ucsd.edu/homer/interactions/, respectively. GITAR is reported in the current study and can be accessed at https://www.genomegitar.org/. TAD, topologically-associated domain.

Here, we present the Genome Interaction Tools and Resources (GITAR), a standardized, easy to use, and flexible solution, to manage Hi-C genomic interaction data, from processing, to storage and visualization. GITAR comprises two modules: HiCtool and the processed data library. All the codes of GITAR are available as open source and the complete pipeline is explicitly provided step-by-step in the documentation. Nothing is left to be programmed by the user, except for parameter tuning to allow full customizability. Therefore, with GITAR, we achieved the goal of building a comprehensive tool, which includes the fundamental steps of the Hi-C data analysis to make it accessible for every type of users, including, for example, experimental biologists or anyone else with no programming or bioinformatic expertise.

## Implementation

### HiCtool: a standardized pipeline to process and visualize Hi-C data

**Figure 1:**
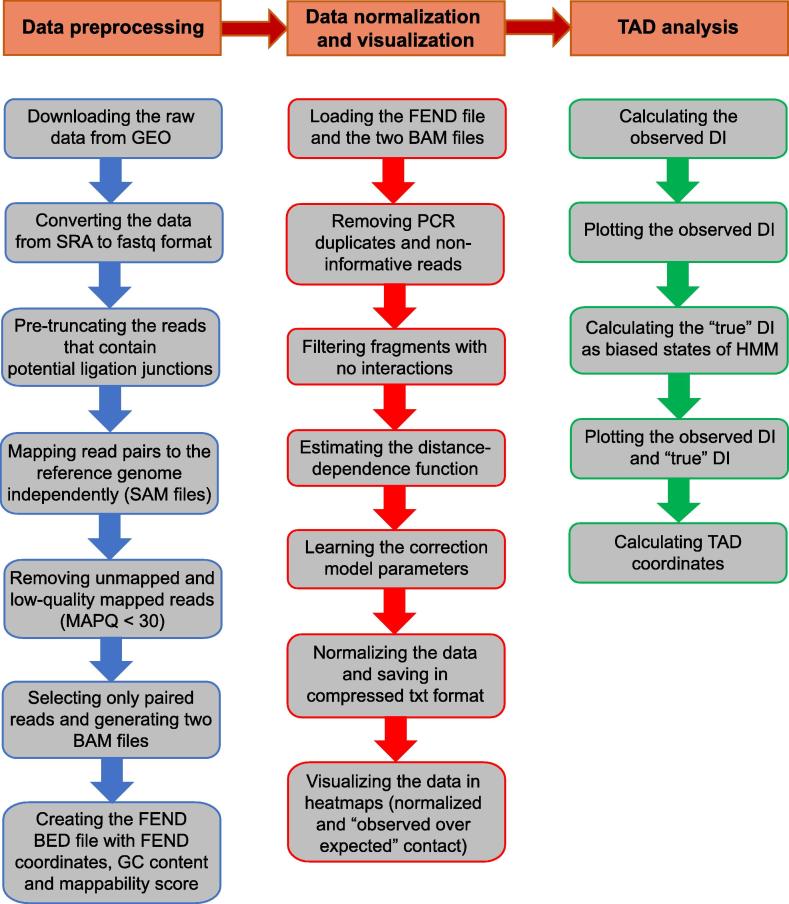
**HiCtool workflow** HiCtool is a pipeline comprising three main sections: data preprocessing, data normalization and visualization (to normalize the contact data and plot heatmaps), and TAD analysis (to calculate the observed DI and “true” DI using HMM, as well as TAD coordinates). TAD, topologically-associated domain; GEO, Gene Expression Omnibus; FEND, fragment end; DI, directionality index; HMM, hidden Markov model; MAPQ, mapping quality.

HiCtool is an open-source bioinformatic tool based on Python, which integrates several software to perform a standardized Hi-C data analysis, from the processing of raw data, to the visualization of intra-chromosomal heatmaps and the identification of TADs. We implemented a pipeline that is divided into three main sections: data preprocessing, data normalization and visualization, and TAD analysis (Figure 1). HiCtool documentation and code are available at http://doc.genomegitar.org.

The data preprocessing pipeline takes files downloaded in SRA format and it requires several steps to generate input files for the normalization procedure. To do so, HiCtool integrates Python code, Unix code, and several software including SRA Toolkit, Bowtie 2, SAMtools, and BEDTools. SRA Toolkit is used for downloading the SRA raw data and conversion to fastq format; then Python code is used to pre-truncate the reads that contain potential ligation junctions as a mapping improvement strategy [10]. Mapping of the read pairs over the reference genome independently is performed subsequently using Bowtie 2 to avoid any proximity constraint; and finally, unmapped and low-quality mapped reads with map quality (MAPQ) <30 are filtered out and only the paired reads are selected using SAMtools and Unix code. Log files are generated at each step for quality control purpose, including number of truncated reads and length distribution plots after pre-truncation, read alignment ratio before and after filtering, and final pairing statistics. At the end, the pipeline enables the generation of the fragment-end (FEND) file used to normalize the data. The FEND file contains restriction site coordinates and additional information such as GC content and mappability score of FENDs [19]. The generation of the FEND file was optimized using parallelized computation to sensibly reduce the time complexity, while we provide the FEND files for human (*hg38*) and mouse (*mm10*) for the most commonly-used restriction enzymes (*Hin*dIII, *Nco*I, *Dpn*II, and *Mbo*I). The outputs of the data preprocessing section include two BAM files corresponding to the first and second reads in the pairs and the FEND file in BED format.

The data normalization and visualization pipeline provides the code to normalize the data and plot the contact heatmaps. The complex experimental Hi-C protocol unavoidably produces several technical biases, which are related to spurious ligation products between fragments, as well as length, GC content, and mappability of the fragments [19]. Moreover, it has been observed that transcription start sites (TSSs) and CTCF binding sites influence the frequency of interactions, due to the local chromatin conformation around them [19].

To normalize the data, we use the Python package HiFive [24], which has been demonstrated before to efficiently handle high-resolution data [24]. HiFive allows to correct all the aforementioned technical biases, taking also into account of “biological biases” that influence the contact frequency (TSSs and CTCF-bound sites), to not confound technical artifacts with meaningful biological features while calculating correction parameters. Before normalization, PCR duplicates and non-informative reads, likely produced due to incomplete restriction enzyme digestion and fragment circularization, were removed. Moreover, HiFive allows to estimate the distance-dependence signal from the data before normalization, thereby avoiding biases caused by the restriction site distribution. Restriction sites are unevenly distributed in the genome, resulting in different distances between fragments and their neighbors. Since the interaction frequency is strongly inversely-related to the inter-fragment distance, fragments surrounded by shorter ones would show higher nearby interaction values than those with longer adjacent fragments [24]. To learn the contact correction parameters associated with fragment features, we use the explicit-factor correction method reported by Yaffe and Tanay [19], and performed by the HiFive binning algorithm, which shows a consistent performance across all binning resolutions [24]. Given that the features in the input FEND file are length, GC content, and mappability, the correction values for contact counts calculated from the model are explicitly related to the cross-correlation of these three factors. “Biological biases” associated with TSSs and CTCF binding sites are considered at this step by excluding fragments interacting within a distance of 500 kb from the model.

After learning the correction parameters, we computed two matrices for each chromosome at a specific bin size. The first is an observed intra-chromosomal contact matrix *O*[*i,j*], where each entry represents the observed read count between the regions identified by the bins *i* and *j*. The second is a correction matrix *E*[*i,j*], where each entry contains the sum of corrections for the read pairs between bins *i* and *j*. Then, the normalized contact matrix *N*[*i,j*] is calculated using the formula:

$$N\left[i,j\right]=\frac{O[i,j]}{E[i,j]}$$

where each entry contains the corrected contact count according to Yaffe and Tanay’s correction model [19]. We computed also the “observed over expected” contact matrix, where the expected counts are calculated considering both the learned correction parameters and the linear distance between read pairs. Specifically, the average intrachromosomal contact probability for loci pairs decreases monotonically with the increase in their linear genomic distance [6].

**Figure 2:**
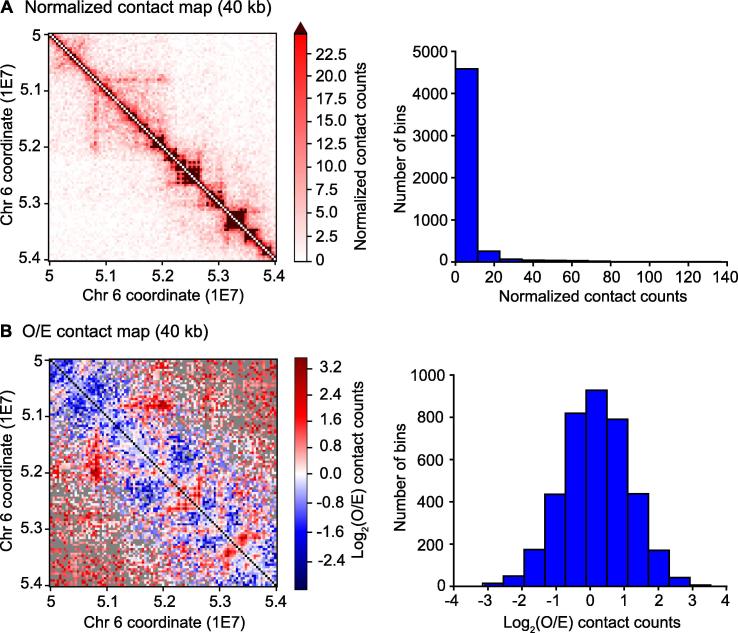
**Contact heatmaps for chromosome 6: [50–54] Mb at the 40-kb resolution** **A.** Normalized contact matrix, where each entry contains the corrected contact count. The upper limit of the colormap is the 95th percentile of the contact counts and values above this limit are represented in brown in the heatmap. Using an upper cut-off for the contact counts allows to emphasize local chromatin structures (TADs and sub-TADs) over the background. The histogram displays the normalized contact count distribution. Values on the X axis indicate the number of contacts, while values on the Y axis indicate the frequency of the bins in the contact matrix with the corresponding contact count on the X axis. The number of contacts ranges from 0 to 137. **B.** O/E contact matrix, where each entry contains the log_2_ of the O/E contact counts, considering the linear distance between loci and the learned correction parameters. Loci without expected contacts and loci without observed contacts are indicated with black and gray pixels, respectively. The histogram displays the O/E contact count distribution in log_2_-transformed form, ranging from −3.153 to 3.530. O/E, observed over expected.

Finally, the pipeline allows to plot the heatmaps, with additional histogram of the data distribution (Figure 2). The intensity of each pixel in the normalized heatmap is the contact count between the corresponding loci (Figure 2A). Normalized data can be plotted either using the full range of contact counts or a cut-off, expressed as a percentile or a maximum number of contacts, to highlight local chromatin structures (such as TADs and sub-TADs) over the background. Complete customizability about the appearance of the maps is provided, by allowing to choose among the default colormaps from Matplotlib or generating a colormap from a custom list of colors. Values above the cut-off are plotted with a different color, which is chosen by the user as well. In addition, the resolution of the heatmap in DPI can be set as well. Increasing the resolution of the figures is critical especially for high resolution contact maps (with low bin sizes), allowing to zoom in while maintaining an optimum visual feedback. For the “observed over expected” (O/E) heatmap, the intensity of each pixel represents the log_2_ of the O/E contact count, to allow easier interpretation of contact enrichment or depletion (Figure 2B). For the visualization functionality, only the Python library Matplotlib was used, without integrating any other external software. Heatmaps and histograms are saved in pdf format.

**Figure 3:**
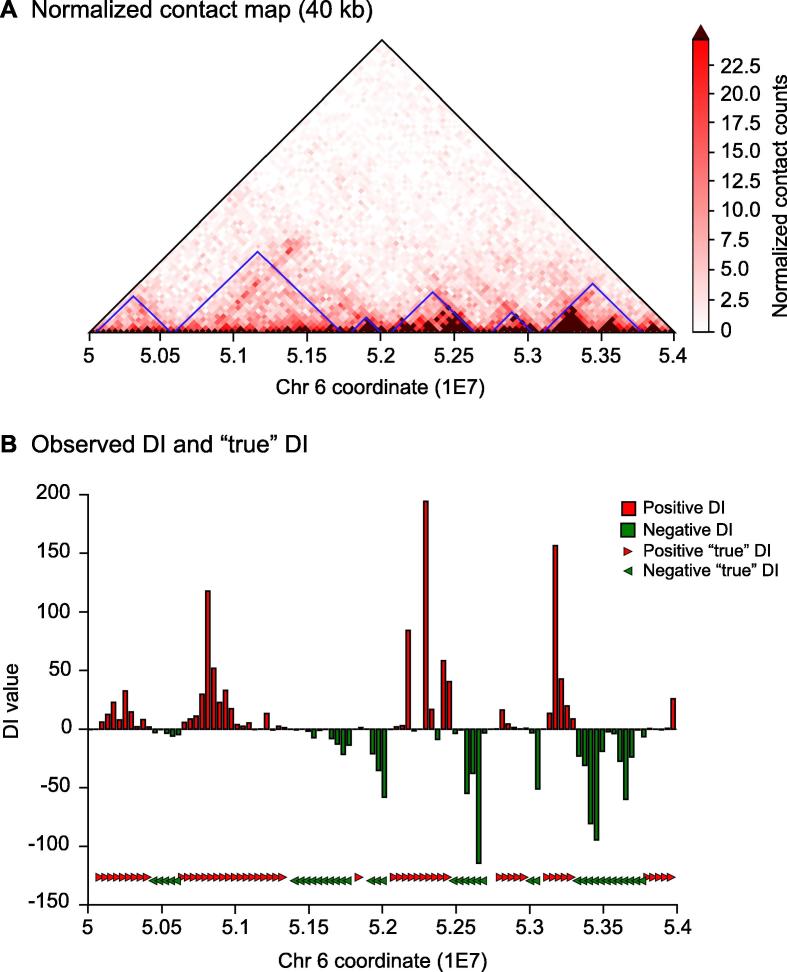
**TADs for chromosome 6: [50–54] Mb at the 40-kb resolution** **A.** Triangular part of the normalized contact matrix. TADs calculated with GITAR are plotted in blue along the diagonal. **B.** Observed DI values and “true” DI values (HMM-biased states). Positive “true” DI and negative “true” DI refer to downstream and upstream HMM-biased states, respectively. HMM-biased states are plotted to show the correspondence with TAD boundaries (Y axis is not informative in this case). According to the HMM state shifts, six TADs and seven TAD boundaries are present. DI, directionality index.

The TAD analysis section provides the code to calculate the directionality index (DI) and the TAD coordinates [8] from the normalized contact data. In order to do so, the observed DI is used to calculate the “true” DI using a hidden Markov model (HMM), implemented with the Python package hmmlearn. Both the observed DI and the “true” DI values can be plotted in the same figure (Figure 3), making it possible to directly infer the presence of TADs and boundaries over the genomic region examined. TAD coordinates are then calculated using the shifts of the HMM-biased states [8]. The entire TAD analysis pipeline is programmed in Python.

### Processed data library

The processed data library is a collection of standardized processed datasets using HiCtool, which is available for the public at http://data.genomegitar.org. So far, we have run 20 datasets of humans (hg38), taken from the library on the 4D Nucleome (4DN) Web Portal (https://4dnucleome.org) [34], and 2 datasets of mice (mm10), to build a large collection that allows consistent comparison among datasets and saving time to process data for visualization or additional analyses. The 4DN library is a collection of genome interaction papers related to the 3C-based assays (3C, 4C, 5C, and Hi-C). Specifically, for GITAR we referred only to Hi-C derived datasets.

Four different outputs for each chromosome of a processed dataset are computed and saved to file: contact matrices, DI values, HMM-biased states (“true” DI values), and TAD coordinates. All the outputs are in txt format, and the functions to load each type of data are provided. Here, we processed the data at 40-kb resolution to perform TAD analysis according to Dixon et al. [8]. In addition, we uploaded to the library the HDF5 files generated by HiFive to allow the user to quickly obtain results at a different resolution, without going through the entire pipeline from the beginning.

Even at the 40-kb resolution, contact matrices contain several million of elements per each chromosome, which requires big storage space, relatively high data saving, and long loading time. To reduce storage usage and computing time, we used an optimized compressed format to store the contact matrices, exploiting their properties of symmetry and sparsity (Figure S1). For instance, if we consider normalized contact matrices for the dataset from Rao et al. [9] (GEO accession No.: GSM1551550) at the 40-kb resolution, our optimized format requires only 13% of space compared to the full contact data (208.6 MB and 1566.7 MB (1.53 GB), respectively). Even if data are compressed into a zip file, our format uses 44% of space for the full data (93.1 MB *vs.* 213 MB). As for the computing time, it takes more than 6 min and 3 min, respectively, to save and load full contact matrices of the aforementioned dataset, whereas saving and loading our parsed data requires less than 2 min for both (hardware: 2.9 GHz Intel Core i5, 16 GB of RAM). At higher resolutions (smaller bin sizes), contact matrices become more sparse; therefore the advantage given by this data format is more remarkable in terms of storage usage and computation time (Table 2).

**Table 2:** **Comparison between full and optimized normalized contact data for the entire human genome**

**Data type**	**Bin size** **(kb)**	**Storage usage** **txt format (MB)**	**Storage usage** **zip format (MB)**	**Saving time** **(min:sec)**	**Loading time**
Full	1000	6.1	2.7	00:01	00:01
Optimized	1000	2.9 (48%)	1.4 (52%)	00:01 (100%)	00:01 (100%)
Full	100	341.4	105.9	01:07	00:30
Optimized	100	110.4 (32%)	49.9 (47%)	00:25 (37%)	00:17 (57%)
Full	40	1566.7	213	06:18	03:24
Optimized	40	208.6 (13%)	93.1 (44%)	01:50 (29%)	01:14 (36%)
Full	10	19,957.8	451.3	90:16	85:41
Optimized	10	393.6 (2%)	177.1 (39%)	26:31 (29%)	18:49 (22%)

*Note*: The dataset was obtained from [9] (GEO accession number: GSM1551550). Hardware: 2.9 GHz Intel Core i5, 16 GB of RAM. The percentage of optimization (optimized/full) at each resolution is indicated in the parentheses. kb, kilobase; MB, megabyte.

Differently from contact matrices, domain coordinates are saved in a format to be read directly. Each line of the txt file refers to a TAD, with tab separated start and end coordinates, allowing quick access and readability.

### Software requirements

The software that are required to run GITAR include Python (>2.7), Bowtie 2, BEDTools, SAMtools, and SRA Toolkit. The Python libraries needed include Numpy, Scipy, Math, Matplotlib, Matplotlib.pyplot, Csv, Pybedtools, Pandas, Multiprocessing (if used), and Biopython. Additionally, Python packages HiFive and Hmmlearn are also needed, with the former used to normalize contact data and the latter serving the HMM to calculate the biased states used to extract TAD coordinates.

## Results

Here, we demonstrate the capability of GITAR to handle high-resolution Hi-C data by processing and visualizing the *in situ* Hi-C dataset GSM1551550. For more details about data processing, refer to File S1.

After raw data are downloaded and converted to fastq format (SRA Toolkit), each of the two fastq files containing paired-end reads is used as input of a Python function, which performs the pre-truncation step. Among the total 202,095,066 read pairs, 29,851,195 (14.78 %) and 28,681,691 (14.20 %) reads, from the first and the second fastq files, respectively, contained a potential ligation junction and were thus truncated. Then, the two pre-truncated fastq files are mapped independently (Bowtie 2) and only reads aligned with MAPQ ≥30 are kept, that is, 172,973,813 (85.59%) and 161,438,783 (79.88%) reads, for the first and the second fastq files, respectively. Finally, only paired reads are selected, thus resulting in a total of 143,415,284 read pairs that go to the normalization pipeline as two separate BAM files, together with the FEND BED file. FEND files for humans (*hg38*) and mice (*mm10*) for the most commonly-used restriction enzymes (*Hin*dIII, *Nco*I, *Dpn*II, and *Mbo*I) are available for direct download; otherwise, any FEND file for a custom species or restriction enzyme can be computed by following our pipeline.

In the normalization pipeline (Python package HiFive [24]), the two BAM files and the FEND file are first used together to generate an HiCData object in HDF5 format, while PCR duplicates, non-informative reads, and spurious ligation products are removed. Then, the HiCData object is loaded into an HiCProject object (HDF5 format), which is used to estimate the distance-dependence signal and run the binning algorithm to perform the correction model for contact counts of Yaffe and Tanay [19]. All these separate steps from HiFive are performed together in our pipeline, by simply using one line of Python code. At this point, normalized contact matrices per each chromosome at a definite bin size can be computed using a single Python function call. To expedite the process of normalization for multiple chromosomes at once, parallelized processing is available through a single Python function as well. Contact maps can be plotted now (Figure 2). The plotting functionality is enclosed in two separate functions: the first can be used to plot observed, expected, and normalized contact matrices (Figure 2A), and the second to plot O/E contact matrices (Figure 2B). Histograms can be produced by setting a boolean parameter in the plotting functions.

Normalized contact matrices at the 40-kb resolution are used to calculate DI values, “true” DI values with HMM and TAD coordinates. DI and “true” DI values are saved to txt file and can also be plotted to infer the presence of TADs in the genomic region under analysis (Figure 3B). TAD coordinates are saved to txt file as well in a tab-separated format. Each of these mentioned tasks is performed in Python using a single function call.

## Conclusion

GITAR is a framework that incorporates all the fundamental steps to process, normalize, visualize, and store Hi-C data in an efficient way. It is freely available as an open-source software at https://genomegitar.org. GITAR consists of two modules: HiCtool and a processed data library. HiCtool is a complete pipeline to work with Hi-C data. It allows to preprocess the raw data, normalize intrachromosomal contact matrices, visualize heatmaps, and also includes TAD analysis. It is built with the aim of making every type of users able to carry out Hi-C data analysis, simply by following a step-by-step documentation available at https://doc.genomegitar.org. Contact matrices are stored in an efficient way to reduce storage occupation and time of I/O operations. The processed data library (https://data.genomegitar.org) is a large collection of processed datasets for different cell lines and conditions of humans and mice, allowing consistent comparison among datasets or further analyses. Future developments of GITAR may include the incorporation of annotation features, additional normalization methods (for example, the scalable matrix balancing method adopted in Hi-Corrector [18]), and expansion of the processed data library with additional datasets.

## Authors’ contributions

RC and SZ conceived the project. RC and QW developed the software and performed the analysis. RC wrote the paper with the contribution of QW and JG. All authors read and approved the final manuscript.

## Competing interests

SZ is a cofounder and a board member of Genemo Inc., which however does not do business related to the work described in this paper.

## Supplementary material

Supplementary File S1 GITAR analysis methods

Supplementary Figure S1 **Contact map storage workflow** A simplified example of the compression workflow, where the intra-chromosomal contact matrix is represented by a 4 × 4 symmetric and sparse matrix. (1) The upper-triangular part of the matrix is selected (including the diagonal); (2) data are reshaped to form a vector; (3) all the consecutive zeros are replaced with a “0” followed by the number of zeros that are repeated consecutively; and (4) data are saved into a txt file.
