# Supplementary material for: GITAR: An Open Source Tool for Analysis and Visualization of Hi-C Data
Source: Genomics Proteomics Bioinformatics. 2018 Dec 13;16(5):365–72. doi: 10.1016/j.gpb.2018.06.006 (PMC6364044; doi:10.1016/j.gpb.2018.06.006)
Supplement: Supplementary File S1 — GITAR analysis methods [file mmc1.docx]

**File S1 GITAR analysis methods**

HiCtool is an open-source software and the source code and documentation are available at [doc.genomegitar.org](http://doc.genomegitar.org/). The preprocessing is based on Python code, Unix code, and several software (SRA Toolkit, Bowtie 2, SAMTools, and BEDTools). The rest of the analysis is programmed in Python, therefore tasks can be performed with single function calls or script executions.

**Mapping**

We performed pre-truncation on reads that contain potential ligation junctions before mapping [10]. All the reads were preprocessed and the ones that contained potential ligation junctions were truncated to keep the longest piece (restriction site sequence included) without the junction sequence. The ligation junction is the concatenation of two filled-in restriction sites (RSs): AAGCTAGCTT for *Hin*dIII, which cuts at A|AGCTT; CCATGCATGG for *Nco*I, which cuts at C|CATGG; GATCGATC for *Mbo*I and *Dpn*II that cut at GATC|. The pre-truncation step was performed using a Python function, where the only inputs required are the fastq files and the restriction enzyme (*Hin*dIII, *Nco*I, *Mbo*I, or *Dpn*II). If a custom restriction enzyme is used, the Python function used to pre-truncate reads allows to input the corresponding ligation junction sequence. A log file is automatically generated with the information about the percentage of reads that have been truncated, and also the length distribution of the truncated reads is plotted in a histogram. Besides pre-truncation, we did not perform any read quality filtering before mapping. After this, mapping was performed using Bowtie 2 with custom parameters, and read pairs were mapped independently on the reference genome to avoid any proximity constraint. After mapping, each fastq file has a corresponding SAM file and log file with statistics of alignment. Then, unmapped reads were discarded and only high quality mapped reads (MAPQ ≥ 30) were kept in each SAM file [19] using SAMtools. After filtering, only paired reads from the SAM files were kept (Unix code) and finally they were converted to BAM format. The two BAM files derived from alignment and processing were not merged because HiFive [24] requires separated BAM files, one per each read of the read pairs.

**Fragment-end file**

The fragment-end (FEND) file is computed by scanning the reference genome for the RSs using Bowtie 2 with the -k flag set to 8000000 (*hg38*: *Hin*dIII, 1.72 million RSs; *Mbo*I, 5.02 million RSs; *mm10*: *Hin*dIII, 1.69 million RSs; *Mbo*I, 2.99 million RSs). The -k argument changes Bowtie 2 alignment behavior. By default, Bowtie 2 searches for distinct, valid alignments for each read. When it finds a valid alignment, it continues to look for alignments that are nearly as good or better, and the best alignment found is reported. When -k <int> is specified, Bowtie 2 searches for at most <int> distinct, valid alignments for each read. The search terminates when it cannot find more distinct valid alignments, or when it finds <int>, if it happens first. In this case, -k 8000000 is assured to cover all the cutting sites throughout the genome, for every restriction enzyme. The alignment produced a SAM file, which was converted to BAM (SAMtools) and then BED format (BEDTtools). In order to correct biases according to Yaffe and Tanay, 2011 [19], information about GC content and sequence uniqueness of the fragments was needed. Given the high time complexity of this job, the pipeline has been optimized for parallelized computation using multiple threads. The first step was to split the FEND file into separated BED files, one per each chromosome. Then, several steps were performed to add first the GC content information, and second the mappability score. The information about the GC percentage was taken from the UCSC table browser (track: GC percent), and saved to separate txt files, one per each chromosome. Specifically, the GC content of the 200 bp upstream and downstream of each RS was calculated, and this was done using 24 parallel threads for *hg38*, one per chromosome. The processor we used was the Xeon E5-2697 v3 (2.60 GHz) and for the FEND file of *Mbo*I on hg38 (5.02 million RSs), the computation time was decreased from 100 h (single thread) to 9 h. To compute the FEND mappability score, the entire genome was split into 50-bp artificial reads starting every 10 bp (Python) to produce a fastq file. The artificial reads were then mapped back to the genome using Bowtie 2. For each FEND, the mappability score was then calculated as the portion of artificial reads mapped with MAPQ > 30 within a 500-bp window upstream and downstream of each RS. Same as for the GC content, this job was done using 24 parallel threads and this reduced the computation time the same (9 h against 100 h with a single thread). After this, FENDs with a mappability score < 0.5 (either upstream or downstream) were discarded.

**Data normalization and visualization**

Data normalization was performed using the Python package HiFive [24]. Both technical biases (spurious ligation products, fragment length, GC content, and mappability score) and biological features, including transcription start sites (TSSs) and CTCF-bound sites, were considered in our pipeline. To remove spurious ligation products, we filtered out paired reads whose total distance from the nearest RSs was > 500 bp. In addition, PCR duplicates were removed and reads with ends mapped to the same fragment and reads with ends mapped to adjacent fragments on opposite strands were also excluded, to consider the possibility of incomplete restriction enzyme digestion and fragment circularization. To take into account of TSSs and CTCF-bound sites, as well as the way they influence the contact frequency upstream and downstream of them, FENDs within a distance of 500 kb are excluded in the learning correction parameter model. This allows to normalize data without confounding technical biases with features associated with biologically-relevant structures. Fragment length, GC content, mappability score, and inter-fragment distance biases are handled and removed using the Binning algorithm, which uses the same probabilistic model as Yaffe and Tanay, 2011 [19]. Fragment length, GC content, mappability score, and inter-fragment distance ranges are divided into 20 bins, such that each bin contains the same number of fragments. For the optimization process of the correction matrices by likelihood maximization, we used a learning threshold of 1 and a maximum number of iterations of 1000.

Heatmaps and histograms were generated and plotted using the Python libraries Matplotlib and Matplotlib.pyplot. The main plotting function allows to plot the observed, expected, and normalized contact matrices, in full or only a portion selected by the user. In addition, a parameter is included for customizing the colormap, either by choosing from one of the styles listed at <https://matplotlib.org/examples/color/colormaps_reference.html> or even by generating a custom one by inserting the colors to be used into a list. Moreover, there is the possibility to select an upper cut-off as a percentile or a maximum value of the contact counts. Values above this cut-off will be plotted in a different color relative to the colormap, which can be chosen by the user as well. The resolution of the heatmap in DPI can be set by tuning a parameter. This allows to zoom in and still maintain a good visual feedback for higher resolution heatmaps (with lower bin sizes). For “observed/expected” (O/E) contact matrices, a separate plotting function is used. In this case, the log_2_(O/E) is plotted to provide a quicker feedback about bins with enrichment or depletion of contact counts.

**Directionality index and TAD computation**

Given a division of the genome into 40 kb bins, we quantified the observed directionality index (DI) using the following formula from Dixon et al., 2012 [8]:

$$DI=\left( \frac{B-A}{\left| B-A \right|} \right) \left( \frac{{(A-E)}^{2}}{E}+ \frac{{(B-E)}^{2}}{E} \right)$$

where A is the number of reads that are mapped from a given 40-kb bin to the upstream 2-Mb region, B is the number of reads that are mapped from a given 40-kb bin to the downstream 2-Mb region, and E is the expected number of contacts for each bin that equals to $\frac{A+B}{2}$. Therefore, the FEND normalized contact data at a bin size of 40 kb is needed to compute the DI. The detection region of 2 Mb for upstream or downstream biases corresponds to 50 bins (2 Mb / 40 kb = 50 bins).

We used a hidden Markov model (HMM) based on the DI to identify biased states. To perform the HMM, we used the Python package hmmlearn. Specifically, we built a model with three biased states corresponding to downstream bias, upstream bias, and no bias, respectively. The sequence of emissions corresponds to the observed DI values, while transition matrix, emission matrix, and initial state sequence are unknown. We have three types of emissions named as 1, 2, and 0 in the model, which correspond to a positive (1), negative (2), or zero (0) value of the observed DI, respectively. In our analysis, we associated the emission ‘0’ with all the absolute DI values under a threshold of 0.4. We initialized transition and emission matrices with the same values of 0.3 for the probabilities to transit to a different state or emission, respectively (values outside the diagonal), and 0.4 for the probabilities of remaining in the same state or observing the same emission (values in the diagonal). So, first we estimated the model parameters and then the most probable sequence of states using the Viterbi algorithm. Biased states were then exploited to calculate topological domain coordinates. According to Dixon and colleagues [8], a domain is initiated at the beginning of a single downstream biased state. The domain is continuous throughout any consecutive downstream biased states and ends when the last in a series of upstream biased states is reached.

**Contact map storage**

HiCtool allows to generate and save contact matrices at the resolution defined by the user. In our pipeline, we processed Hi-C data with a bin size of 40 kb to allow topological domain analysis [8]. Even at this resolution, contact matrices contain several million of elements per each chromosome, requiring big storage space and relatively high data saving and loading time. To address this problem, we proposed a way to parse the data based on the fact that contact maps are symmetric (contacts between loci *i* and *j* are the same than those between loci *j* and *i*) and usually sparse, since most of the elements are zeros, and this property is stronger with the decrease in the bin size. Given these two properties, it is not needed to save mirrored data and moreover it would be useful to “compress” the zero data within the matrices. To accomplish this, first we selected only the upper-triangular part of the contact matrices (including the diagonal) and reshaped the data by rows to form a vector. After that, we replaced all the consecutive zeros in the vector with a “0” followed by the number of zeros that are repeated consecutively; all the non-zero elements are left as they are. Finally, the data are saved in a txt file (Figure S1). As mentioned before, at higher resolutions (low bin sizes) contact matrices show more zeros, meaning that the advantage given by this data format would be more remarkable in terms of storage usage and computation time (Table 2).

**Software requirements**

The software that are required to use GITAR include Python (> 2.7), Bowtie 2, BEDTools, SAMTools, and SRA Toolkit. The Python libraries needed include Numpy, Scipy, Math, Matplotlib, Matplotlib.pyplot, Csv, Pybedtools, Pandas, Multiprocessing (if used), and Biopython. Additionally, Python packages HiFive and Hmmlearn are also needed, with the former used to normalize contact data and the latter serving the HMM to calculate the biased states used to extract topological domain coordinates.
