# Supplementary figures and images for: GITAR: An Open Source Tool for Analysis and Visualization of Hi-C Data
Source: Genomics Proteomics Bioinformatics. 2018 Dec 13;16(5):365–72. doi: 10.1016/j.gpb.2018.06.006 (PMC6364044; doi:10.1016/j.gpb.2018.06.006)

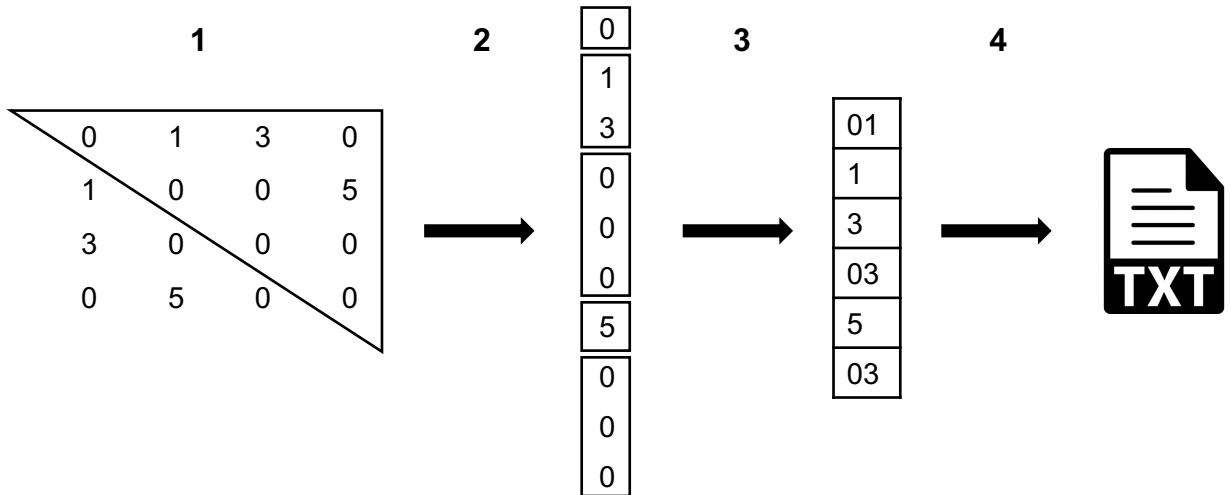

Supplement: Supplementary Figure S1 — Contact map storage workflow A simplified example of the compression workflow, where the intra-chromosomal contact matrix is represented by a 4 × 4 symmetric and sparse matrix. (1) The upper-triangular part of the matrix is selected (including the diagonal); (2) data are reshaped to form a vector; (3) all the consecutive zeros are replaced with a “0” followed by the number of zeros that are repeated consecutively; and (4) data are saved into a txt file. [file mmc2.pdf]
